# Is Static Alignment a Good Predictor of Dynamic Alignment after Total Knee Arthroplasty?

**DOI:** 10.3390/healthcare10030418

**Published:** 2022-02-23

**Authors:** Cheng Gu, Yurong Mao, Haiyan Dong, Yu Cui, Ming Fu

**Affiliations:** 1 Department of Joint Surgery, The First Affiliated Hospital, Sun Yat-sen University, Guangzhou 510080, China; guch8@mail2.sysu.edu.cn; 2 Guangdong Provincial Key Laboratory of Orthopedics and Traumatology, The First Affiliated Hospital, Sun Yat-sen University, Guangzhou 510080, China; 3 Department of Rehabilitation Medicine, The First Affiliated Hospital, Sun Yat-sen University, Guangzhou 510080, China; maoyr@mail.sysu.edu.cn; 4 Department of Rehabilitation Medicine, The Seventh Affiliated Hospital, Sun Yat-sen University, Shenzhen 518107, China; 5 Guangdong Provincial Key Laboratory of Colorectal and Pelvic Floor Diseases, Guangdong Institute of Gastroenterology, The Sixth Affiliated Hospital, Sun Yat-sen University, Guangzhou 510655, China; donghy23@mail2.sysu.edu.cn; 6 School of Life Sciences, The Chinese University of Hong Kong, Hong Kong 999077, China; cuiyu@link.cuhk.edu.hk

**Keywords:** arthroplasty, knee joint, alignment, osteoarthritis, gait analysis

## Abstract

Background: Total knee arthroplasty (TKA) is the only effective treatment of end-stage knee osteoarthritis (OA). Lower limb neutral alignment has been a criterion to predict prosthesis life; however, there has been recent controversy over this. Some researchers believe that lower limb static alignment does not significantly affect prosthesis life and some researchers have found that dynamic mechanical alignment may affect prosthesis life, which needs to be further studied. Methods: Eighty-seven patients with knee OA were evaluated by a three-dimensional (3D) gait analysis system before TKA and six months after TKA, dynamic mechanical alignment and basic gait parameters were then calculated. Based on the static alignment of the lower limb on the postoperative X-radiographs, they were divided into a neutral alignment group (58 cases), varus alignment group (20 cases), and valgus alignment group (9 cases). Simple linear regression was used to assess the correlation between static and dynamic alignment. One-way analysis of variance (ANOVA) was used to compare the differences in gait parameters between and within groups. Results: Eighty-seven patients were followed up for an average of six months after the operation. There was no significant difference in all gait parameters among the three groups after TKA. There was no correlation found between static alignment and dynamic alignment/knee adduction moment (KAM) after TKA, although patients showed a significant linear correlation before operation. There was a significant linear correlation between dynamic alignment and KAM before and after the operation. Conclusions: Static alignment has no significant effect on postoperative gait function. Static alignment is no longer an effective predictor of the dynamic alignment or KAM six months after TKA, although they are correlated before TKA. The dynamic alignment allows for better prediction of KAM, which may be a risk factor for the life of the prosthesis.

## 1. Introduction

The data show that the incidence rate of knee OA is about 16−17%, and the peak is usually around 75 years old, reflecting a younger trend [1]. TKA, an essential method for treating end-stage knee OA, has shown promising clinical results. It is reported that the number of patients who received TKA has exceeded 600,000 every year in the United States; this number is still growing rapidly. It is estimated that the primary TKA will reach 3.4 million cases per year by 2030 [2]. According to reports, the 10-year survival rate of prostheses in the primary TKA is 93.3%, and the 25-year survival rate is about 82% [3,4]. Despite the continuous improvement of surgical techniques and prosthesis types, some TKA patients still suffer from persistent pain or dysfunction after surgery, which affects their postoperative satisfaction and prosthesis life [5].

Normal lower limb alignment is from the center of the femoral head to the center of the ankle joint; this line passes through the center of the knee joint or is slightly medially deviated [6]. It deviates from the neutral vertical by approximately 0±3° and can be divided into the femoral mechanical axis, which is the connection between the center of the femoral head and the distal intercondylar fossa of the femur, and the tibial mechanical axis, which is the connection between the proximal tibia and the center of the ankle joint. The angle formed between them is called the hip–knee–ankle angle (HKA), which can represent lower limbs’ mechanical alignment. The lower limb mechanical alignment of patients with knee OA is often accompanied by severe varus and valgus. Therefore, TKA needs to reconstruct the lower limb mechanical alignment. At present, many studies have shown that it is reasonable to ensure HKA in the neutral (0±3°) range after TKA [7,8]. Beyond this range, patients may suffer postoperative discomfort and shorten the life of prosthesis [9]. At present, the gold standard of surgery is still to achieve 0±3°neutral mechanical alignment [10]. Werner et al. [11] found that when HKA is controlled in the neutral position, the medial and lateral gaps of the knee joint are roughly equal. Townley et al. [12] proposed that the best alignment position for the lower limb mechanical alignment after TKA should be slightly varus because the load by the medial compartment of the knee joint of the average human body is greater than that of the lateral compartment. However, Matziolis et al. [13] found that the deviation of lower limb mechanical alignment after TKA has no apparent association with postoperative dysfunction. Increasingly more studies [14] have proven that the static alignment after TKA does not sufficiently reflect the prosthesis life.

Gait analysis is a biomechanical research method used to analyze the functional state of human walking by using the principles of motion mechanics, human anatomy, and physiology. The process of the human body movement, based on a complex movement model, according to the model theory, allows the lower limbs to be recorded and quantitatively analyzed [15]. In the 1960s, 3D gait analysis changed from a simple qualitative description to a quantitative analysis in kinematics and has been widely used in clinical practice [16]. Significantly, the 3D motion capture system of the Vicon is an advanced and objective gait evaluation method. The development and progress of 3D gait analysis systems have been an important research direction in orthopedics, rehabilitation, neurology, and other fields [17,18,19].

Previous gait studies have confirmed that knee adduction moment (KAM) represents the medial compartment load of the knee joint [20]. Its calculation method is the ground reaction force multiplied by the distance between the force point and the joint center, which is the primary basis for the knee joint’s change of medial and lateral load [12]. So far, KAM has been recognized as a good substitute for the medial compartment load of the knee, and excessive KAM is closely related to the occurrence and progression of knee OA [19,20,21,22]. The imbalance of load distribution between medial and lateral compartments is considered to be a risk factor affecting the life of prosthesis [11,20]. Wasielewski et al. found that imbalance load between the medial and lateral compartments may lead to abnormal wear of the polyethylene insert and prosthesis, which may affect the life of the prosthesis or cause prosthesis loosening [23]. However, whether postoperative KAM is related to lower limb alignment is controversial [14,24]. Therefore, whether the static mechanical alignment of lower limbs can predict the dynamic mechanical alignment and KAM remains to be further studied.

We hypothesize that static alignment is not a good predictor of dynamic alignment and KAM, and dynamic alignment can be a predictor of KAM and prosthesis life.

The goal of this study was to characterize the gait parameters before and six months after TKA, to evaluate the correlation among static alignment, dynamic alignment and KAM, and explore the predictability of static and dynamic mechanical alignment for KAM and prosthetic life.

## 2. Methods

### 2.1. Participants

This is a prospective study of knee OA patients who received TKA. Patients’ demographic information was collected, including age, gender, and BMI before operation. 3D gait analysis was performed before surgery and about six months after surgery. From February 2021 to September 2021, knee OA patients were recruited in this study. Participants were included when they met all of the following criteria: (1) patient had been diagnosed with knee OA and scheduled for primary TKA; (2) patient had agreed to participate in our study; (3) patient could walk 10 m unassisted; (4) patient had been measured for 3D gait motion analysis; and (5) patient was able to cooperate with postoperative follow-up. Exclusion criteria included: (1) patient had a history of a knee operation; (2) patient had surgical history of the hip or ankle; (3) a lack of X-ray image data; (4) no TKA or postoperative loss of follow-up; and (5) there were adverse events such as prosthesis infection, fracture, and dislocation. The preoperative 3D gait movement analysis included one hundred and two patients, but eight were lost to follow-up, two received knee single condylar replacement instead of TKA, three patients lacked X-ray photos of lower limbs after the operation, and two had not received surgery for other reasons. Eighty-seven patients were finally included. The institutional ethics committee of the First Affiliated Hospital of Sun Yat-sen University approved the study for clinical research, and each participant provided informed consent.

### 2.2. Surgery and Clinical Assessment

**Figure 1:**
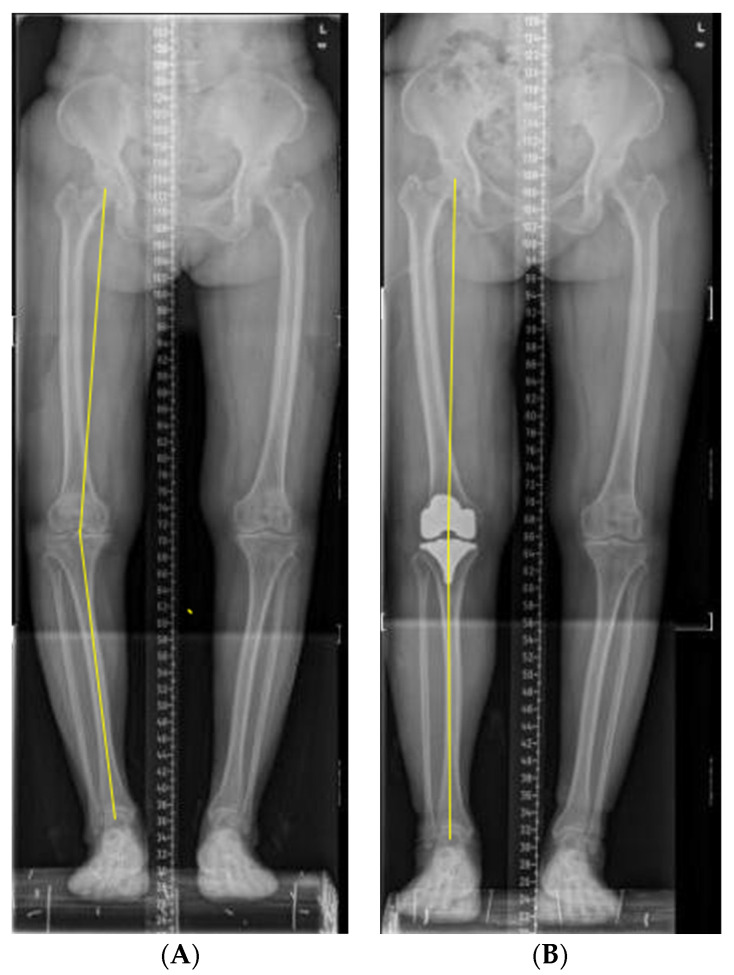
The static lower limb alignment was measured on the radiograph. (**A**) Before operation; (**B**) after operation.

These operations were performed by doctors in the same department. The prosthesis selection was based on the patient’s knee joint’s soft tissue and mechanical line. The surgical approaches were anterior median incision of the knee, medial parapatellar approach, tibial bone marrow external positioning, femoral bone marrow internal positioning, and a posterior reference system. The operation method measured the soft tissue balance according to the flexion and extension space after osteotomy. All surgical procedures sought optimal mechanical alignment. Intravenous or oral analgesics were obtained, standardized anticoagulation, and routine knee rehabilitation training was carried out. The X-ray was rechecked in time after operation to ensure the prosthesis was in place. The X-ray was evaluated for HKA reflecting limb static mechanical alignment, as shown in Figure 1. HKA is defined as 0° when two axes are on a straight line, and HKA is defined as positive in varus and negative in valgus. The 87 patients were divided into three groups according to static HTA. There were 58 cases of the neutral-aligned group (0 ± 3°), 20 cases of varus-aligned type (>3° varus), and 9 cases of valgus-aligned type (>3° valgus). Patients’ satisfaction was scored using the Western Ontario and McMaster University Osteoarthritis Index (WOMAC). The WOMAC scale was used to evaluate pain levels before and six months after the operation in this study. If the patient has no pain or functional limitation, the score is 0 points, and the score of extreme pain or functional limitation is 100 points. The scale has been proven to be closely related to the recovery of knee function after operation [25].

### 2.3. Gait Analysis

**Figure 2:**
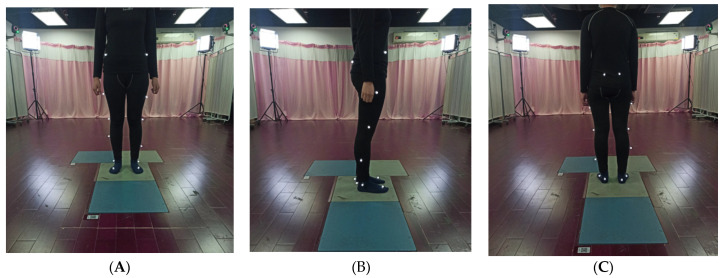
The marker model is part of the 3D gait analysis procedure. (**A**) Front view; (**B**) lateral view; (**C**) opposite view.

**Figure 3:**
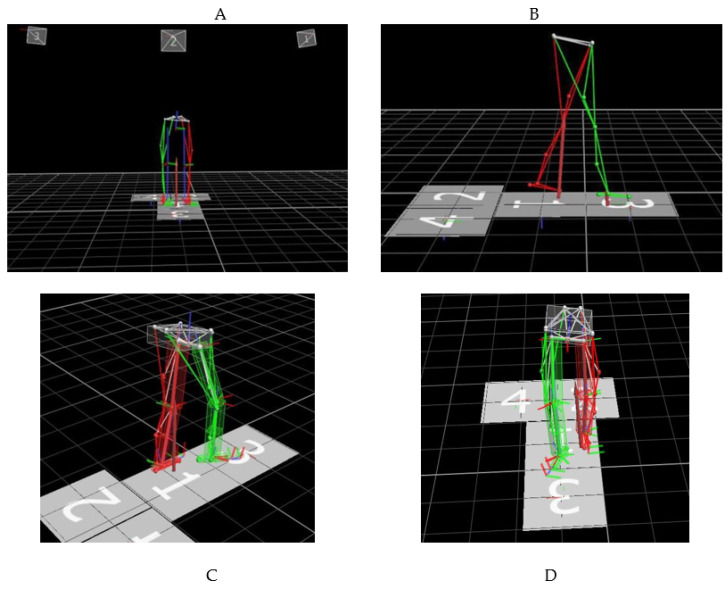
Gait analysis with VICON system. (**A**) Static model; (**B**) patient gait acquisition; (**C**) and (**D**) 3D view after dynamic model.

**Figure 4:**
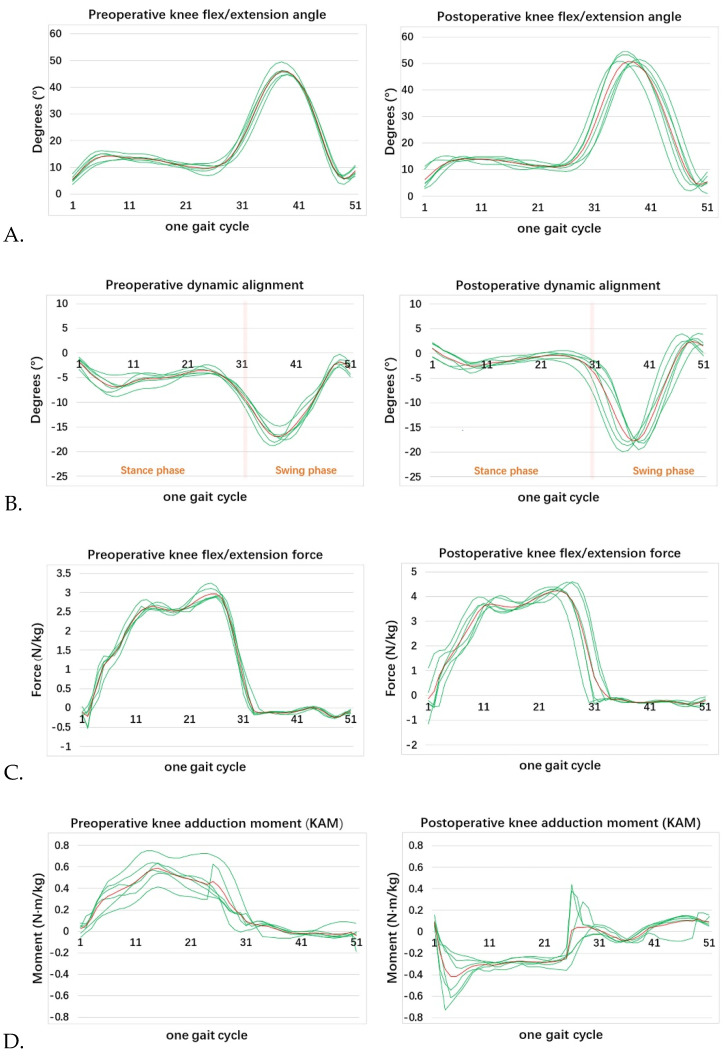
Some gait parameters of the same patient before and after TKA. The six green lines represent the dynamic parameter change of each gait, and the red line represents the average readings of six gait measurements. (**A**) Knee flexion/extension angle; (**B**) Dynamic alignment angle; (**C**) Knee flexion/extension force and (**D**) Knee adduction moment (KAM).

3D kinematic data were collected utilizing a Vicon 3D motion capture system (Vicon, Oxford, UK), containing six infrared cameras and four force plates. Infrared cameras were used to capture kinematic and spatiotemporal parameters [14]. Force platforms were used to collect the kinetic parameters [26]. Sixteen retroreflective markers were placed on bony anatomical landmarks of the lower extremity. The marker set included bilateral anterior superior iliac spine, and bilateral posterior superior iliac spine, lower lateral 1/3 and 1/2 surface of the left and right thigh, bilateral lateral knee joint lines, lower lateral 1/3 and 1/2 surface of the left and right calves, bilateral lateral malleoli, bilateral heels, and bilateral heads of the second metatarsals [27,28]. Figure 2 shows a scene where one of the patients used a Vicon 3D gait analyzer for marker calibration. First, we measured the height, weight, length of the lower limbs (distance between anterior superior iliac spine and medial malleolus), width of knee, and width of ankle. Next, we input these data into the computer to establish basic information of subjects, and the marker balls were pasted strictly following the specified parts. Then, as shown in Figure 3, we asked subjects to stand on force plates and collect the static calibration trial. Finally, subjects were told to walk in their usual way; the subjects were encouraged to walk several times and get familiar with the test process before starting the formal test. First, the static model of the subject was established, and then the subject walked straight on the test track according to the usual walking mode and speed to ensure that each foot stepped on a force measuring platform. Then, six gaits with good image quality were selected for dynamic modeling. Nexus software was used to analyze the measured data. Finally, the six experimental gait data were averaged for subsequent analysis. The gait parameters include moment, force, movement angle, and basic gait parameters such as cadence, stride time, stride length, single/double support, and speed, as shown in Figure 4. The dynamic mechanical alignment is the mean value of the coronal alignment of the lower limb during the stance phase. Dynamic HKA reflected the dynamic mechanical alignment of lower limbs. The measurement of dynamic HKA was the same as that of static HKA, it was also positive in varus, negative in valgus, and 0° in neutral position.

### 2.4. Statistical Analysis

Statistical analyses above were performed using the Statistical Package for the Social Sciences (SPSS v.26; SPSS Inc. Chicago, IL). The measurement data are expressed as mean ± standard deviation (x ± sd). Simple linear regression was used to evaluate the association between static mechanical alignment, dynamic mechanical alignment, and KAM after TKA, removal of outliers with significant deviation and the correlation was tested by Fisher’s exact test. ANOVA compares the demographic characteristics and gait parameters of three groups. Post-hoc multiple comparisons were used for least significance difference (LSD). The difference is statistically significant when *p* < 0.05.

## 3. Results

### 3.1. Patient Characteristics

A total of 87 patients were evaluated preoperatively and about six months postoperatively. Table 1 presents the baseline characteristics of three groups. There was no significant difference in age, height, weight, BMI, and HKA variation among the three groups (*p* > 0.05). HKA variation was calculated as difference between preoperative HKA and postoperative HKA. Table 2 represents the 3D gait parameters and WOMAC score before TKA. There was no significant difference among the three groups. In Table 3, there was also no significant difference in these parameters among the three groups six months after TKA. Paired sample *t*-test was used to compare preoperative and postoperative WOMAC score. The result showed that there was significant difference in WOMAC score before and after operation, and the WOMAC score decreased significantly after operation, suggesting that TKA significantly improved knee function.

### 3.2. Association between Static Mechanical Alignment and Dynamic Alignment

**Figure 5:**
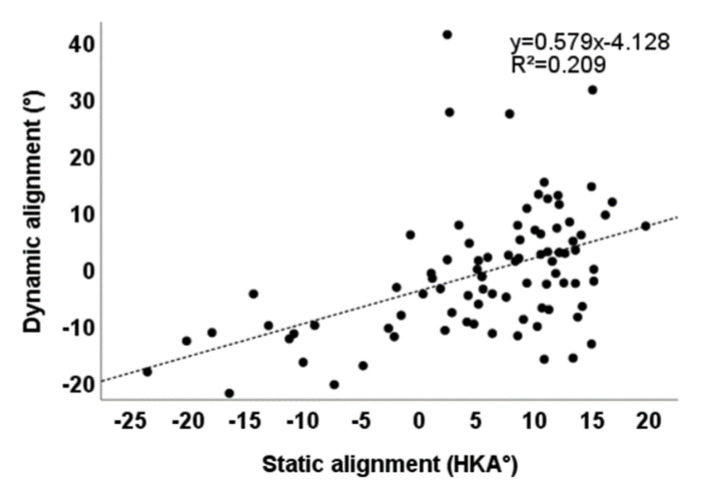
Correlation between static alignment and dynamic alignment before operation (*p* < 0.001).

**Figure 6:**
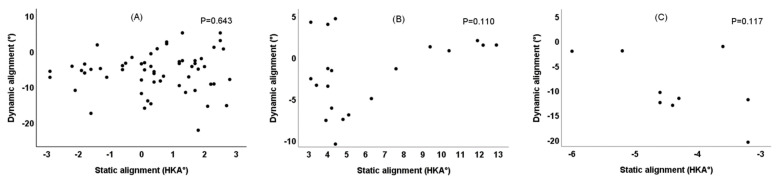
Correlation between static alignment and dynamic alignment six months after TKA. (**A**) Neutrally-aligned group; (**B**) varus-aligned group; (**C**) valgus-aligned group.

There was a significant linear correlation between static mechanical alignment and dynamic mechanical alignment before TKA in 87 patients with knee OA (R^2^ = 0.209, *p* < 0.001) (Figure 5). After TKA, there was no correlation between static mechanical alignment and dynamic mechanical alignment in the three groups (Figure 6).

### 3.3. Association between Mechanical Alignment and KAM

**Figure 7:**
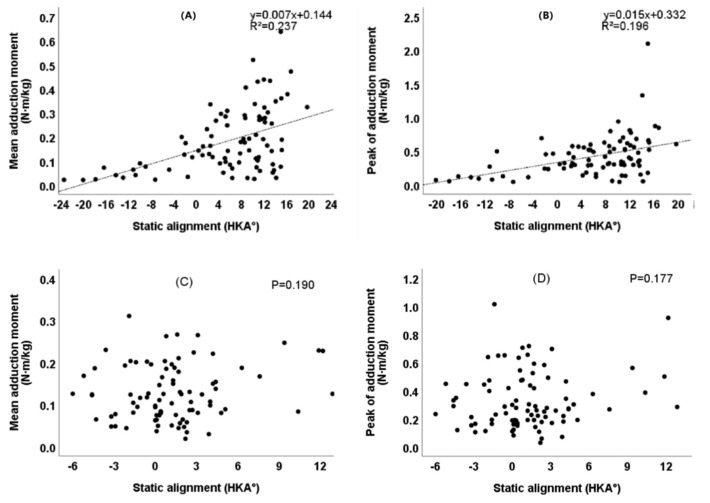
Correlation between static alignment and KAM six months after TKA. (**A**) Correlation between preoperative static alignment and mean KAM (*p* < 0.001). (**B**) Correlation between preoperative static alignment and peak of KAM (*p* < 0.001). (**C**) Correlation between postoperative static alignment and mean KAM. (**D**) Correlation between postoperative static alignment and peak of KAM.

**Figure 8:**
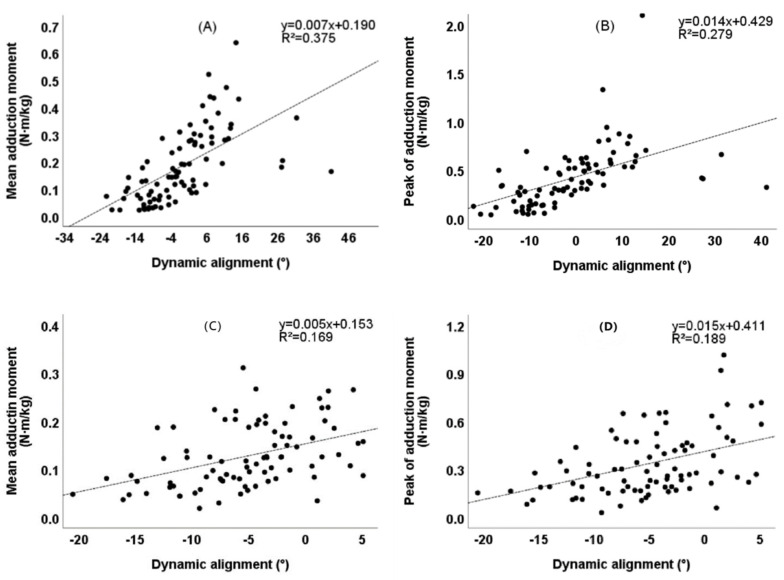
Correlation between dynamic alignment and knee KAM. (**A**) Correlation between preoperative dynamic alignment and mean KAM (*p* < 0.001). (**B**) Correlation between preoperative dynamic alignment and peak of KAM (*p* < 0.001). (**C**) Correlation between postoperative dynamic alignment and mean KAM (*p* < 0.001). (**D**) Correlation between postoperative dynamic alignment and peak of KAM (*p* < 0.001).

As shown in Figure 7, there was a significant linear correlation between the preoperative static mechanical alignment and the mean KAM/peak of KAM (R^2^ = 0.237, *p* < 0.001)/ (R^2^ = 0.196, *p* < 0.001), and the postoperative static mechanical alignment lost correlation with the mean KAM/peak of KAM (*p* = 0.190, *p* = 0.177). As shown in Figure 8, there was a significant linear correlation between the preoperative dynamic mechanical alignment and the mean KAM/peak of KAM (R^2^ = 0.375, *p* < 0.001)/ (R^2^ = 0.279, *p* < 0.001), and this correlation still existed after the operation (R^2^ = 0.169, *p* < 0.001)/ (R^2^ = 0.189, *p* < 0.001).

## 4. Discussion

TKA is an effective method for the treatment of advanced knee OA. Most patients can obtain good clinical results after operation. However, 20% of patients still fail to achieve satisfactory results, often characterized by pain and dysfunction [29]. Previous studies have found that postoperative functional recovery is affected by many factors, and there is even an interaction between some factors. The gait analysis based on an infrared camera and force measuring platform is the gold standard for accurate gait analysis. The Vicon system used in this study is the most classic 3D gait analysis system [30]. At present, there are still many gaps in the research of 3D gait analysis after TKA. Using a Vicon gait analysis system to quantify the relevant factors affecting knee function will help surgeons make better clinical decisions and improve the clinical efficacy of TKA.

One of the operation principles of TKA is to realize the neutral alignment of lower limbs, which is generally considered to be 0±3°, to avoid abnormal wear of the prosthesis and the discomfort of patients. In our experiment, six months after operation patients were divided into the neutral-aligned group, varus-aligned group, and valgus-aligned group according to the static alignment of their lower limbs. The gait characteristics and WOMAC score of the three groups were compared. It was found that there was no difference among the three groups, suggesting that the alignment of lower limbs may have no significant effect on the dynamic function of the knee joint. More studies suggest that the postoperative neutral alignment might not be better than another alignment [31,32].

The lower limb alignment measured by postoperative X-ray photos is static mechanical alignment, while people are often active, and the knee joint is also a moving joint. The wear of prosthesis and patient discomfort often occurs in the dynamic process of walking rather than when in static position. Therefore, postoperative dynamic alignment has more clinical value and prediction function than static alignment. We take for granted that when in postoperative static neutral alignment, the dynamic alignment should also be neutral, but this may not be the case [33]. Therefore, this experiment studied the correlation between preoperative and postoperative static mechanical alignment and dynamic mechanical alignment. It was found that there was a significant linear correlation between preoperative static alignment and dynamic alignment (Figure 5). However, there was no correlation between postoperative static alignment and dynamic alignment (Figure 6), indicating that TKA surgery might change this correlation between static alignment and dynamic alignment. The static alignment of postoperative X-ray is challenging to predict its dynamic alignment; this is consistent with the results of Mündermann et al. [34]. This may be because the soft tissue release in pursuit of neutral alignment during TKA surgery changes the balance of the knee joint. Studies have shown that during gait, the relaxation of the medial muscles and soft tissue leads to abnormal gait and function, while the relaxation of the lateral joint is tolerable to a certain extent [35,36]. Therefore, special attention should be paid to maintaining the stability of medial soft tissue during TKA. Statistics show that [37], using mechanical alignment technology, 25% of varus knees and 54% of valgus knees will produce an imbalance of more than 3 mm on the medial and lateral gap, resulting in a smaller lateral gap and larger medial gap, increasing the imbalance of soft tissue. Mechanical alignment changes the natural joint line and causes ligament imbalance and abnormal gait, so it needs intraoperative soft tissue balance.

We further studied the correlation between lower limb alignment and KAM. Whether before or after TKA, we found that there was a significant linear correlation between the dynamic alignment and KAM (Figure 8). This shows that the pressure changes of the medial and lateral compartments of the joint are probably affected by the dynamic alignment. The dynamic alignment might have more predictive value for the wear and life of the prosthesis after TKA. In the preoperative patients, the static alignment was significantly linearly correlated with KAM (Figure 7), indicating that the more varus before operation, the greater the load of the medial compartment of the knee joint, which is consistent with the research results of Morishima et al. [33]. There was no correlation between the postoperative static alignment and KAM, indicating that the postoperative static alignment might have difficulties to reflect the medial and lateral compartments load of the joint during movement. That may explain why many studies have found there to be no correlation between the static alignment and the life of the knee prosthesis [38].

There are limitations in this experiment: (1) The sample size was small in the valgus group; thus, more cases are required in future studies. (2) The follow-up time was only about 6 months, which may lead to deviation in the results; more time points of follow-up are required for understanding the long-term outcomes. (3) The postoperative gait characteristics are closely related to the postoperative rehabilitation training; patients’ postoperative rehabilitation training varies greatly, which may be the interference factor leading to the postoperative gait difference.

## 5. Conclusions

In summary, the most important finding of this study is that there was found to be no correlation between static alignment and dynamic alignment/KAM after TKA. However, a significant linear correlation was found before operation. Moreover, there was no difference in gait characteristics and WOMAC score among patients with static neutral, varus, and valgus alignment. This suggests that the postoperative static alignment has no effect on gait functions, and static alignment may not be a reliable factor to evaluate dynamic alignment and KAM after TKA. The dynamic alignment may better reflect the medial and lateral compartments load of the knee joint, which might be a predictor for the life of the prosthesis.

## Figures and Tables

**Table 1:** Demographic characteristics of the knee OA patients who received TKA (n = 87) (Mean ± standard deviation).

Variables	Neutral Alignment Group (n = 58)	Varus Alignment Group (n = 20)	Valgus Alignment Group (n = 9)	*p* Value
				ANOVA
Age (years)	68.64 ± 8.10	70.95 ± 5.96	64.22 ± 9.30	0.104
Gender, males/females	14/44	5/15	0/9	-
Height (cm)	158.84 ± 6.98	156.85 ± 8.22	158.00 ± 5.20	0.557
Weight (kg)	66.22 ± 10.79	61.75 ± 8.08	62.94 ± 8.45	0.196
BMI (kg/m^2^)	26.19 ± 3.62	25.13 ± 2.96	25.20 ± 3.04	0.413
Surgical side, right/left	39/19	12/8	3/6	-
Postoperative static HKA (°)	0.46 ± 1.51	6.17 ± 3.31	−4.34 ± 0.92	<0.001
Postoperative dynamic HKA (°)	−5.88 ± 5.64	−1.88 ± 4.40	−9.49 ± 6.47	0.002
Static HKA variation (°)	3.63 ± 9.19	5.26 ± 4.97	6.01 ± 10.90	0.623
Dynamic HKA variation (°)	4.96 ± 13.77	2.95 ± 7.37	4.09 ± 13.58	0.824

**Table 2:** 3D gait parameters and WOMAC score of the knee OA patients before TKA (n = 87) (Mean ± standard deviation).

Variables	Neutral Alignment Group (n = 58)	Varus Alignment Group (n = 20)	Valgus Alignment Group (n = 9)	*p* Value
				ANOVA
Cadence (step/min)	87.14 ± 15.84	92.09 ± 13.95	88.32 ± 21.98	0.498
Stride time (s)	1.43 ± 0.29	1.35 ± 0.25	1.45 ± 0.42	0.497
Opposite foot off (%)	14.72 ± 3.82	13.50 ± 2.69	15.04 ± 4.53	0.394
Opposite foot contact (%)	49.44 ± 2.46	48.86 ± 2.34	49.49 ± 1.72	0.632
Step time (s)	0.73 ± 0.16	0.69 ± 0.12	0.73 ± 0.20	0.609
Single support (s)	0.49 ± 0.10	0.48 ± 0.10	0.50 ± 0.18	0.823
Double support (s)	0.42 ± 0.16	0.36 ± 0.11	0.42 ± 0.15	0.393
Foot off (%)	63.21 ± 3.50	62.05 ± 3.37	62.87 ± 2.60	0.429
Stride length (m)	0.72 ± 0.20	0.77 ± 0.21	0.64 ± 0.20	0.241
Step length (m)	0.34 ± 0.11	0.38 ± 0.11	0.32 ± 0.11	0.321
Walking speed (m/s)	0.53 ± 0.21	0.60 ± 0.21	0.48 ± 0.23	0.291
Dynamic range of motion (°)	32.22 ± 14.89	35.00 ± 19.47	28.95 ± 18.04	0.636
Extension moment (N·m/kg)	0.29 ± 0.18	0.46 ± 0.59	0.27 ± 0.17	0.110
Internal rotation moment (N·m/kg)	0.05 ± 0.04	0.53 ± 2.06	0.05 ± 0.02	0.163
Extension force (N/kg)	1.33 ± 0.58	1.69 ± 0.90	1.20 ± 0.43	0.074
Maximum flexion angle (°)	36.83 ± 17.28	38.63 ± 12.69	37.14 ± 18.13	0.915
WOMAC score	53 ± 17	53 ± 14	46 ± 11	0.473

**Table 3:** 3D gait parameters and WOMAC score of the knee OA patients six months after TKA (n = 87) (Mean ± standard deviation).

Variables	Neutral Alignment Group (n = 58)	Varus Alignment Group (n = 20)	Valgus Alignment Group (n = 9)	*p* Value
				ANOVA
Cadence (step/min)	92.42 ± 11.19	92.38 ± 8.41	92.26 ± 11.99	0.999
Stride time (s)	1.32 ± 0.17	1.31 ± 0.12	1.32 ± 0.17	0.969
Opposite foot off (%)	11.91 ± 2.02	11.48±1.75	10.93 ± 0.84	0.294
Opposite foot contact (%)	49.78 ± 1.62	49.08±1.93	50.05 ± 1.76	0.218
Step time (s)	0.66 ± 0.09	0.67±0.65	0.66 ± 0.08	0.975
Single support (s)	0.50 ± 0.05	0.49±0.45	0.52 ± 0.07	0.491
Double support (s)	0.31 ± 0.08	0.31 ± 0.61	0.29 ± 0.36	0.757
Foot off (%)	61.02 ± 2.15	60.85 ± 1.78	61.05 ± 2.03	0.950
Stride length (m)	0.75 ± 0.16	0.72 ± 0.14	0.69 ± 0.20	0.570
Step length (m)	0.37 ± 0.08	0.36 ± 0.76	0.33 ± 0.13	0.484
Walking speed (m/s)	0.57 ± 0.15	0.56 ± 0.11	0.53 ± 0.15	0.594
Dynamic range of motion (°)	15.41 ± 9.04	15.03 ± 9.55	16.07 ± 13.60	0.964
Extension moment (N·m/kg)	0.26 ± 0.16	0.28 ± 0.16	0.26 ± 0.13	0.880
Internal rotation moment (N·m/kg)	0.03 ± 0.02	0.04 ± 0.02	0.04 ± 0.01	0.526
Extension force (N/kg)	1.02 ± 0.62	1.06 ± 0.64	1.08 ± 0.60	0.946
Maximum flexion angle (°)	37.09 ± 16.21	33.45 ± 11.11	40.06 ± 13.17	0.490
WOMAC score	15 ± 10	16 ± 8	12 ± 5	0.494

## Data Availability

The data presented in this study are available on request from the corresponding author. The data are not publicly available due to involves the privacy of patients’ personal gait data.

## References

[1] Hunter D.J., Bierma-Zeinstra S. (2019). Osteoarthritis. Lancet.

[2] Dreyer H.C., Strycker L.A., Senesac H.A., Hocker A.D., Smolkowski K., Shah S.N., Jewett B.A. (2013). Essential amino acid supplementation in patients following total knee arthroplasty. J. Clin. Investig..

[3] Niinimäki T., Eskelinen A., Mäkelä K., Ohtonen P., Puhto A.P., Remes V. (2014). Unicompartmental knee arthroplasty survivorship is lower than TKA survivorship: A 27-year Finnish registry study. Clin. Orthop. Relat. Res..

[4] Evans J.T., Walker R.W., Evans J.P., Blom A.W., Sayers A., Whitehouse M.R. (2019). How long does a knee replacement last? A systematic review and meta-analysis of case series and national registry reports with more than 15 years of follow-up. Lancet.

[5] Degen R.M., Matz J., Teeter M.G., Lanting B.A., Howard J.L., McCalden R.W. (2018). Does Posterior Condylar Offset Affect Clinical Results following Total Knee Arthroplasty?. J. Knee Surg..

[6] Jeffery R.S., Morris R.W., Denham R.A. (1991). Coronal alignment after total knee replacement. J. Bone Jt. Surg. Br. Vol..

[7] Hetaimish B.M., Khan M.M., Simunovic N., Al-Harbi H.H., Bhandari M., Zalzal P.K. (2012). Meta-analysis of navigation vs conventional total knee arthroplasty. J. Arthroplast..

[8] Fu Y., Wang M., Liu Y., Fu Q. (2012). Alignment outcomes in navigated total knee arthroplasty: A meta-analysis. Knee Surg. Sports Traumatol. Arthrosc..

[9] Cherian J.J., Kapadia B.H., Banerjee S., Jauregui J.J., Issa K., Mont M.A. (2014). Mechanical, Anatomical, and Kinematic Axis in TKA: Concepts and Practical Applications. Curr. Rev. Musculoskelet. Med..

[10] Larose G., Fuentes A., Lavoie F., Aissaoui R., de Guise J., Hagemeister N. (2019). Can total knee arthroplasty restore the correlation between radiographic mechanical axis angle and dynamic coronal plane alignment during gait?. Knee.

[11] Werner F.W., Ayers D.C., Maletsky L.P., Rullkoetter P.J. (2005). The effect of valgus/varus malalignment on load distribution in total knee replacements. J. Biomech..

[12] Mandeville D., Osternig L.R., Lantz B.A., Mohler C.G., Chou L.S. (2008). The effect of total knee replacement on the knee varus angle and moment during walking and stair ascent. Clin. Biomech..

[13] Matziolis G., Adam J., Perka C. (2010). Varus malalignment has no influence on clinical outcome in midterm follow-up after total knee replacement. Arch. Orthop. Trauma Surg..

[14] Miller E.J., Pagnano M.W., Kaufman K.R. (2014). Tibiofemoral alignment in posterior stabilized total knee arthroplasty: Static alignment does not predict dynamic tibial plateau loading. J. Orthop. Res..

[15] Boudarham J., Roche N., Pradon D., Bonnyaud C., Bensmail D., Zory R. (2013). Variations in kinematics during clinical gait analysis in stroke patients. PLoS ONE.

[16] Baker R. (2007). The history of gait analysis before the advent of modern computers. Gait Posture.

[17] Wang J., Severin A.C., Mears S.C., Stambough J.B., Barnes C.L., Mannen E.M. (2021). Changes in Mediolateral Postural Control Mechanisms During Gait After Total Knee Arthroplasty. J. Arthroplast..

[18] Syczewska M., Szczerbik E., Kalinowska M., Swiecicka A., Graff G. (2021). Are Gait and Balance Problems in Neurological Patients Interdependent? Enhanced Analysis Using Gait Indices, Cyclograms, Balance Parameters and Entropy. Entropy.

[19] Khalaj N., Abu Osman N.A., Mokhtar A.H., Mehdikhani M., Wan Abas W.A. (2014). Effect of exercise and gait retraining on knee adduction moment in people with knee osteoarthritis. Proc. Inst. Mech. Engineers. Part H J. Eng. Med..

[20] Niki Y., Nagura T., Nagai K., Kobayashi S., Harato K. (2018). Kinematically aligned total knee arthroplasty reduces knee adduction moment more than mechanically aligned total knee arthroplasty. Knee Surg. Sports Traumatol. Arthrosc..

[21] Manal K., Buchanan T.S. (2022). An Efficient One-Step Moment Balancing Algorithm for Computing Medial and Lateral Knee Compartment Contact Forces. J. Biomech. Eng..

[22] Milner C.E., O’Bryan M.E. (2008). Bilateral frontal plane mechanics after unilateral total knee arthroplasty. Arch. Phys. Med. Rehabil..

[23] Wasielewski R.C., Galante J.O., Leighty R.M., Natarajan R.N., Rosenberg A.G. (1994). Wear patterns on retrieved polyethylene tibial inserts and their relationship to technical considerations during total knee arthroplasty. Clin. Orthop. Relat. Res..

[24] Halder A., Kutzner I., Graichen F., Heinlein B., Beier A., Bergmann G. (2012). Influence of limb alignment on mediolateral loading in total knee replacement: In vivo measurements in five patients. J. Bone Jt. Surg..

[25] Jeong H.S., Lee S.C., Jee H., Song J.B., Chang H.S., Lee S.Y. (2019). Proprioceptive Training and Outcomes of Patients With Knee Osteoarthritis: A Meta-Analysis of Randomized Controlled Trials. J. Athl. Train..

[26] Maier M.W., Aschauer S., Wolf S.I., Dreher T., Merle C., Bitsch R.G. (2019). Three dimensional gait analysis in patients with symptomatic component mal-rotation after total knee arthroplasty. Int. Orthop..

[27] D’Anchise R., Andreata M., Balbino C., Manta N. (2013). Posterior cruciate ligament-retaining and posterior-stabilized total knee arthroplasty: Differences in surgical technique. Joints.

[28] Mullaji A.B., Shetty G.M. (2013). Surgical technique: Computer-assisted sliding medial condylar osteotomy to achieve gap balance in varus knees during TKA. Clin. Orthop. Relat. Res..

[29] Carr A.J., Robertsson O., Graves S., Price A.J., Arden N.K., Judge A., Beard D.J. (2012). Knee replacement. Lancet.

[30] Bonnefoy-Mazure A., Favre T., Praplan G., Armand S., Sagawa Junior Y., Hannouche D., Turcot K., Lübbeke A., Miozzari H.H. (2020). Associations between gait analysis parameters and patient satisfaction one year following primary total knee arthroplasty. Gait Posture.

[31] Rivière C., Ollivier M., Girerd D., Argenson J.N., Parratte S. (2017). Does standing limb alignment after total knee arthroplasty predict dynamic alignment and knee loading during gait?. Knee.

[32] Parratte S., Pagnano M.W., Trousdale R.T., Berry D.J. (2010). Effect of postoperative mechanical axis alignment on the fifteen-year survival of modern, cemented total knee replacements. J. Bone Jt. Surg..

[33] Orishimo K.F., Kremenic I.J., Deshmukh A.J., Nicholas S.J., Rodriguez J.A. (2012). Does total knee arthroplasty change frontal plane knee biomechanics during gait?. Clin. Orthop. Relat. Res..

[34] Mündermann A., Dyrby C.O., Andriacchi T.P. (2008). A comparison of measuring mechanical axis alignment using three-dimensional position capture with skin markers and radiographic measurements in patients with bilateral medial compartment knee osteoarthritis. Knee.

[35] Ushio T., Mizu-Uchi H., Okazaki K., Miyama K., Akasaki Y., Ma Y., Nakashima Y. (2019). Medial soft tissue contracture does not always exist in varus osteoarthritis knees in total knee arthroplasty. Knee Surg. Sports Traumatol. Arthrosc..

[36] Tsukiyama H., Kuriyama S., Kobayashi M., Nakamura S., Furu M., Ito H., Matsuda S. (2017). Medial rather than lateral knee instability correlates with inferior patient satisfaction and knee function after total knee arthroplasty. Knee.

[37] Blakeney W., Beaulieu Y., Puliero B., Kiss M.O., Vendittoli P.A. (2020). Bone resection for mechanically aligned total knee arthroplasty creates frequent gap modifications and imbalances. Knee Surg. Sports Traumatol. Arthrosc..

[38] Gao Z.X., Long N.J., Zhang S.Y., Yu W., Dai Y.X., Xiao C. (2020). Comparison of Kinematic Alignment and Mechanical Alignment in Total Knee Arthroplasty: A Meta-analysis of Randomized Controlled Clinical Trials. Orthop. Surg..

